# Novel TLR7 hemizygous variant in post-COVID-19 neurological deterioration: a case report with literature review

**DOI:** 10.3389/fneur.2023.1268035

**Published:** 2023-11-29

**Authors:** Ahmed Noor Eddin, Mohammed Al-Rimawi, Feham Peer-Zada, Khalid Hundallah, Amal Alhashem

**Affiliations:** ^1^College of Medicine, Alfaisal University, Riyadh, Saudi Arabia; ^2^Division of Pediatric Neurology, Department of Pediatrics, Prince Sultan Medical Military City, Riyadh, Saudi Arabia; ^3^Division of Genetic and Metabolic Medicine, Department of Pediatrics, Prince Sultan Medical Military City, Riyadh, Saudi Arabia

**Keywords:** COVID-19, SARS-CoV-2, TLR7, hemizygous, neurological deterioration, immunodeficiency, genetics, case report

## Abstract

The neurological complications of coronavirus disease 2019 (COVID-19) can range from simple tremors and dystonia to features of encephalopathy. Toll-like receptor 7 (TLR7) belongs to a family of innate immune receptors responsible for viral RNA detection (such as SARS-CoV-2) and immune response initiation. TLR7 loss of function variants have been previously reported as genetic risk factors for severe COVID-19 infection in young patients with no comorbidities. In this case, we report a pediatric patient who developed severe long-term neurological deterioration following his COVID-19 infection. Presenting first to the clinic with episodic dystonia and finger spasticity, the patient’s condition rapidly deteriorated with a significant drop in the Glasgow Coma Scale (GCS). Despite improvement following initial treatment with rituximab and intravenous immunoglobulin, the patient’s symptoms relapsed, and GCS further dropped to 3/15. Serial brain magnetic resonance imaging scans revealed diffuse parenchymal atrophy, ventricular enlargement, and spinal cord thickening. Autoimmune investigations were negative but clinical whole genome sequencing prioritized four gene variants, the most significant of which was a novel frameshift null variant of the X chromosomal TLR7 gene (c.1386_1389dup, p.[His464Ilefs*7]). This case illustrates a role for TLR7 in long-term COVID-19 complications and highlights that TLR7 deficiency in the future may be addressed as a therapeutic measure.

## 1 Introduction

Beginning as a local outbreak in China, the coronavirus disease 2019 (COVID-19) pandemic grew to have profound global impact, lasting over 2 years and afflicting more than 600 million people worldwide (1). Although most patients infected with the severe acute respiratory syndrome coronavirus 2 (SARS-CoV-2) represent asymptomatic or mild cases (~80%), some may develop severe pneumonia (~15%) or life-threatening acute respiratory distress syndrome (ARDS) (~5%) (2). Well-established risk factors that predispose patients to more severe forms of COVID-19 include sex (male), advanced age (greater than 60 years), and previous comorbidity (namely hypertension and diabetes) (3). Critical disease, however, has also been reported in previously healthy young patients and, thus, it is likely that genetic factors mediating viral entry or underlying a host’s immune response may be at play. These factors may also help explain the long-term effects or complications some patients develop after infection resolution. Recent evidence suggests that variants of the X-linked Toll-like receptor 7 (TLR7) gene may help predict COVID-19 severity and penetrance in younger patients (4, 5). Herein, we report a novel, likely pathogenic variant of TLR7 in a pediatric patient with severe neurological deterioration following COVID-19 infection. We also comprehensively review any reported literature on TLR7 variants in COVID-19 patients.

## 2 Case presentation

**Figure 1:**
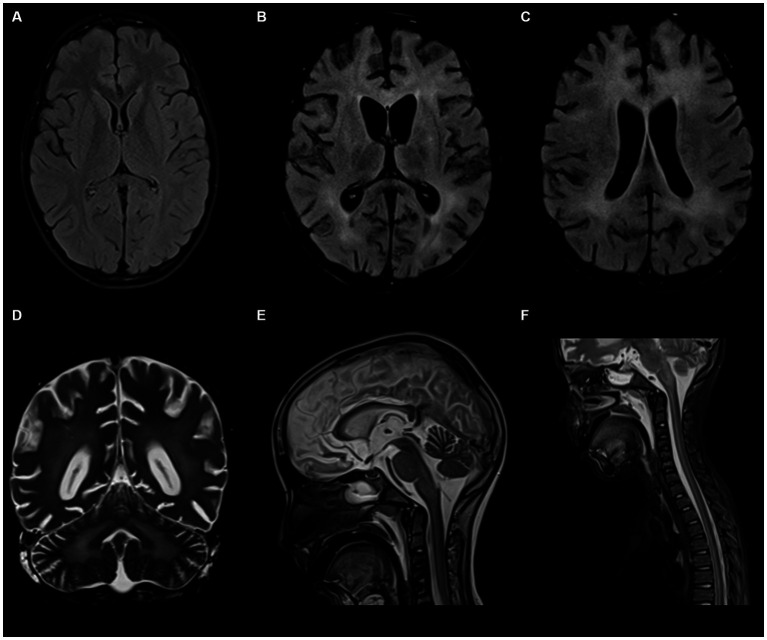
Brain and spinal cord magnetic resonance imaging (MRI) scans of the 10-year-old patient. **(A)** Represents the first MRI taken of the patient after his initial presentation: unremarkable with no sign of acute or remote brain insult. **(B–D)** Represent the second set of MRIs taken approximately 5 weeks since the patient’s initial visit: abnormal symmetrical T2 and FLAIR white matter high signal intensities are noted in the middle cerebellar peduncles, pons, and cortices with focal atrophic changes and compensatory ventricular enlargement. **(E,F)** Represent contrast-enhanced MRIs of brain and spinal cord: abnormal diffuse T2 signal intensities involving the central and anterior cord extending down to the conus medullaris.

The patient is a previously healthy 10-year-old boy, born to consanguineous parents in a twin pregnancy, who presented to the pediatric clinic of his home city with ataxia, left arm dystonia, and brief, episodic neck flexion to the left side. The patient’s past medical history was uneventful, except for a moderate COVID-19 infection with respiratory symptoms like cough and fever that resolved within two weeks prior to the clinic visit. There are no similar symptoms in the patient’s twin brother, and he is not known to have any medical illnesses. Family history revealed that the parents were first-degree cousins and had no history of similar problems of seizures or autoimmune diseases in the family (see Supplementary Figure S1 for pedigree). The patient was administered sodium valproate, along with one course of prophylactic intravenous immunoglobulin (IVIG) for suspected auto-immune encephalitis. He was subsequently discharged, but no improvement was observed. In fact, his symptoms progressed over two weeks to difficulty in speech and swallowing, tongue involvement, and drooling of saliva, and the patient was admitted to the hospital. On physical exam, spastic tone and brisk reflexes without clonus were noted. Comprehensive laboratory assessments, including basic labs, liver function test (LFT), C-reactive protein (CRP), coagulation profile, ammonia and lactate were within normal range. Notably, thyroglobulin antibodies were increased 4-fold (16.5 IU/mL), raising suspicion of Hashimoto’s encephalitis. However, when antithyroid peroxidase antibodies and anti-thyroglobulin antibodies were repeated in our hospital, both antibodies were negative. Brain computed tomography (CT) and magnetic Resonance Imaging (MRI) scans were performed, but the results were unremarkable (see Figure 1A). Electroencephalogram (EEG) and sleep graphs revealed abnormal time-logged periodic complex with sharp theta paroxysm seen every 4–5 s, suggesting progressive myoclonic epilepsy as a possible differential. Nevertheless, when given multiple anti-epileptic drugs including Clonazepam (0.5 mg PO TID), Carbidopa-Levodopa (75 mg AM, 50 mg noon, 75 mg PM, PO, TID), Benzotropine (2 mg PO OD) and Baclofen (5 mg PO TID), there was still no improvement.

Therefore, for further evaluation, the patient was transferred to the Pediatric Intensive Care Unit (PICU) in our hospital. On re-examination, the patient continued to exhibit frequent dystonic repetitive movements now associated with tachycardia and desaturation. Excessive drooling, along with spasticity in both upper and lower limbs, hyperreflexia across all tendons, and positive clonus, were also noted. The Glasgow Coma Scale (GCS) score was 8/15. To address the patient’s deteriorating vital signs and secure the airway, intubation was deemed necessary. The intervention notably improved the tachycardia. Furthermore, the administration of a Midazolam infusion (4 mic/kg/min) and fentanyl (2 mic/kg/h) effectively halted the dystonic episodes, resulting in a temporary improvement in the GCS score to 11/15. To confirm the placement of the endotracheal tube, a chest X-ray was taken, which showed significant perihilar peri-bronchial wall thickening and interstitial infiltrates, thought to be a sequelae of his moderate COVID-19 infection. Blood, urine, respiratory, and cerebrospinal fluid (CSF) cultures were obtained, but the results were negative one week later. In light of the absence of infectious markers, the suspicion of autoimmune encephalitis (AE) following a prior COVID-19 infection was raised. Nevertheless, the autoimmune serology panel was negative for dsDNA, ANA, cANCA, and pANCA antibodies with normal levels of anti-MPO (1.39) and anti-PR3 (2.67) antibodies were detected (see Supplementary Table S1).

On routine examination five days after the patient’s intubation, 3 mm sluggish pupils were noted bilaterally and GCS had decreased again to 8/15. The patient exhibited hypertonia in both upper and lower limbs, and the gag and cough reflexes remained intact. Another EEG was performed, revealing diffuse slowing of electrical activity. Brain MRI was repeated the following day, demonstrating new development of anterior and superior bifrontal periventricular, deep and subcortical T2 and fluid-attenuated inversion recovery (FLAIR) white matter high signal intensity, focal atrophic changes and compensatory widening of the ventricular system (see Figures 1B–D). The visualized upper cervical spinal cord also showed faint high T2 signal intensity within the spinal cord. These findings are suggestive of progressive multifocal leukoencephalopathy (PML) with brainstem and spinal cord involvement in addition to mild brain volumetric loss. Results of the CSF analysis three days later revealed high CSF total protein (0.37 mmol/L), high CSF IgG/albumin ratio (2.67), low serum albumin levels (26 g/L), and the detection of a CSF albumin oligoclonal band, collectively suggesting an active inflammatory state that may contribute to the patient’s declining condition (see Supplementary Table S2). A blood sample was taken and sent for whole exome sequencing (WES). For the next two weeks, the patient was given one course of Rituximab (490 mg and 356 mg), three courses of IVIG (>27 g) and received a two-day steroid pulse therapy.

Prior to immunotherapy, the patient had spiked a fever of 38.6°C, which resolved. Following immunotherapy treatment and vital stability, he was discharged from the PICU with a central line. However, four days later, he was readmitted with a low-grade fever of 38.1°C. Blood and urine cultures were negative, while the respiratory culture was positive for Methicillin-resistant *Staphylococcus aureus* (MRSA). The patient was given tazocin and vancomycin was added after nine days. However, his symptoms persisted, with recurrent low-grade fevers, tachypnea, and desaturation despite multiple changes of antibiotics. The patient required re-intubation. Subsequent extubation attempts proved unsuccessful, even in the absence of any respiratory infection.

**Figure 2:**
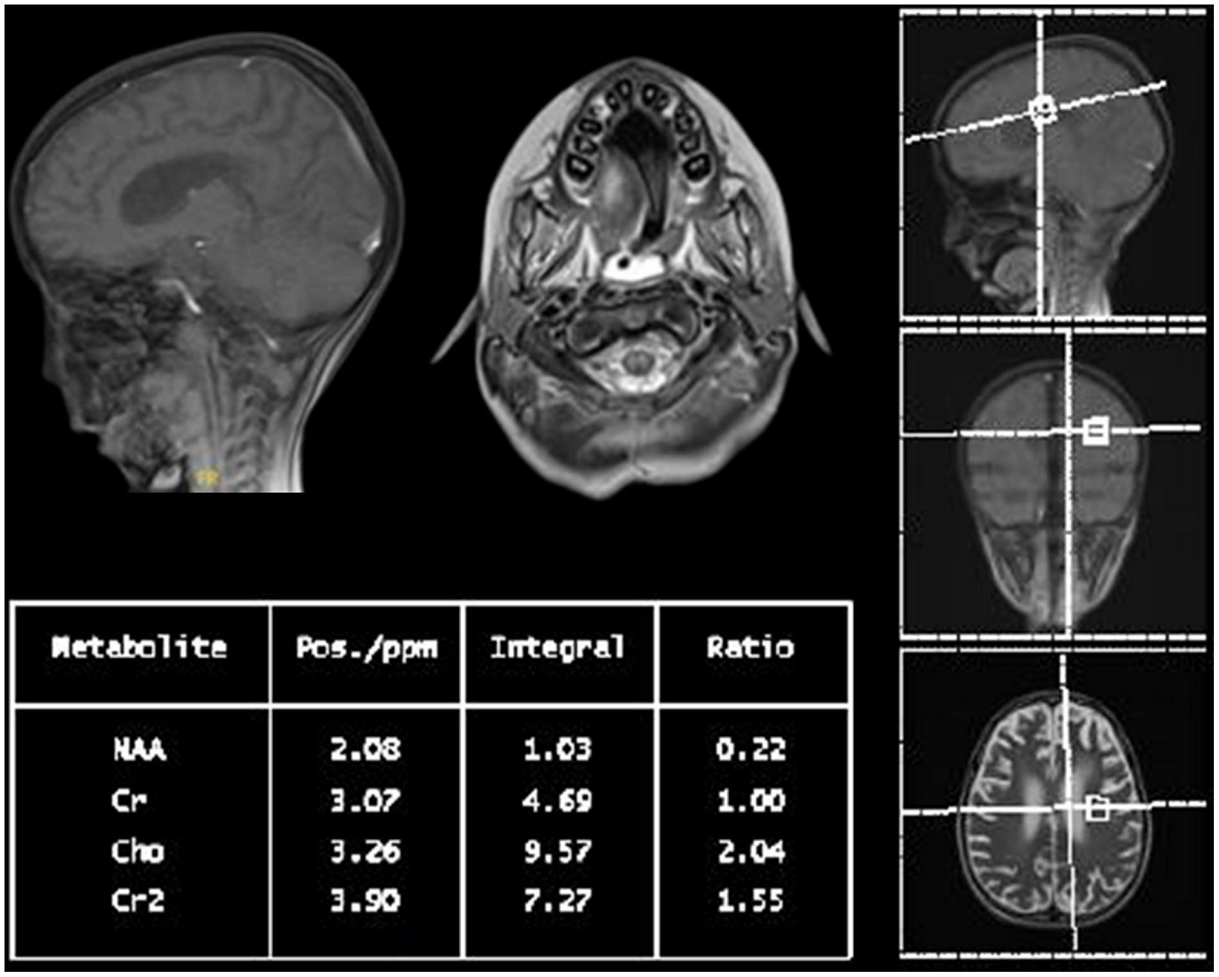
Multi-voxel MR spectroscopy of the patient: short and long TE at the basal ganglia, peritrigonal white matter, and cerebellar peduncles with increased choline to creatinine ratio and a decreased N-acetylaspartate to creatine ratio suggest an underlying inflammatory or demyelinating process.

Within a month, the patient’s GCS significantly deteriorated to 3/15. Pupils were fixed and dilated at 6 mm, the gag reflex disappeared, and the corneal reflex exhibited minimal response. Clonus, which was previously present, was now absent. WES results returned negative, revealing no clinically relevant variants related to the described phenotype. This ruled out the differential diagnosis of progressive myoclonic epilepsy as well as other potential metabolic or mitochondrial diseases that could account for the patient’s symptoms. Two weeks later, with no improvement and the absence of the corneal reflex, a buccal swab sample was sent for whole genome sequencing (WGS). No further escalation of immune therapy occurred, and only symptomatic treatment for seizures was provided. Brain MRI indicated further worsening of the diffuse brain parenchymal atrophy with resultant ventricular enlargement while spinal MRI revealed abnormal diffuse T2 signal intensities involving the central and anterior cord extending down to the conus medullaris (see Figures 1E,F). Brain multi-voxel MR spectroscopy findings were further suggestive of an inflammatory or demyelinating process post-COVID-19 infection (see Figure 2).

The clinical WGS one month later prioritized four gene variants. A hemizygous, likely pathogenic variant of the TLR7 gene was detected (chrX:12886892, NM_016562.4: c.1386_1389dup; p.His464Ilefs*7) consistent with the genetic diagnosis of X-linked recessive COVID-19-related immunodeficiency type 74. Three additional variants were identified: homozygous, likely pathogenic variant of MEFV Innate Immunity Regulator, Pyrin (MEFV) gene (chr16:3243257, NM_000243.3:c.2230G > T; p.Ala744Ser), consistent with the genetic diagnosis of autosomal recessive Familial Mediterranean Fever; homozygous, variant of uncertain significance of Sodium Voltage-Gated Channel Alpha Subunit 5 (SCN5A) gene (chr3:38604004, NM_001099404.2:c.1598G > A; p.Arg533His), suggesting autosomal dominant Sick Sinus Syndrome type 1; and lastly heterozygous variant of uncertain significance of Signal Transducer and Activator of Transcription 3 (STAT3) gene (chr17:42337519, NM_139276.3:c.713A > C; p.Glu238Ala), indicating possible autosomal dominant infantile-onset multisystem autoimmune disease. The pathogenicity and clinical impact of the variants in each of these genes are detailed in Table 1. Thus, a diagnosis of neurological deterioration post-COVID-19 due to TLR7 deficiency was made.

**Table 1:** Summary of pathogenicity, type, and clinical presentation of the major genetic variants detected.

Gene	TLR7 gene	MEFV gene	SCN5A gene	STAT3 gene
Physiologic function	Recognition of pathogen-associated molecular patterns (PAMPs) and activation of innate immunity	Receptor for autophagic degradation of inflammasomes and regulation of IL1B- or IL18-mediated inflammation	Integral membrane protein in cardiac myocytes responsible for the initial upstroke of the action potential on electrocardiogram	Signal transducer that translocates to the nucleus and activates transcription for cell growth, proliferation, or apoptosis
ID transcript	NM_016562.4	NM_000243.3	NM_001099404.2	NM_139276.3
Patient variant*	c.1386_1389dup, p.(His464Ilefs*7)	c.2230G > T, p.(Ala744Ser)	c.1598G > A, p.(Arg533His)	c.713A > C, p.(Glu238Ala)
Variant type	Nonsense frameshift, premature stop codon causing loss of function	Missense, alanine to serine at codon 744	Missense, arginine to histidine at codon 533	Missense, glutamine to alanine at codon 238
Variant zygosity	Hemizygous	Homozygous	Homozygous	Heterozygous
Variant pathogenicity	Likely pathogenic (class 2)	Uncertain significance (class 3)	Uncertain significance (class 3)	Uncertain significance (class 3)
Mode of inheritance	X-linked recessive	Autosomal recessive	Autosomal dominant	Autosomal dominant
Associated condition	COVID-19-related immunodeficiency type 74	Familial mediterranean fever	Congenital sick sinus syndrome type 1	Infantile-onset multisystem autoimmune disease
Clinical features	Severe respiratory insufficiency following COVID infection with defective type I and II interferon (IFN) responses	Recurrent transient episodes of serositis which, if untreated, may cause amyloidosis and subsequent renal failure	Syncope, pre-syncope, dizziness, and fatigue but may occasionally cause tachycardia-bradycardia syndrome	Early onset of insulin-dependent diabetes mellitus, autoimmune enteropathy, celiac disease, or autoimmune hematologic disorders
Clinical impact	High	Moderate	Moderate	Moderate

*Sequence reads of each sample were mapped to the human reference genome (hg38).

Unfortunately, the prognosis for TLR7 deficiency remains challenging, as there is currently no specific treatment available. The patient was repeatedly admitted to the ICU, without significant improvement. His neurological symptoms, including reduced deep tendon reflexes and increased dystonia, have progressively worsened. The patient complained of constitutional symptoms due to recurring fevers and infections which were symptomatically treated. Two months later, despite stable vital signs and physical tests, the patient suffered a cardiac arrest. He was resuscitated and has been in static condition since. The patient exhibits no spontaneous breathing and remains ventilator-dependent. A concise but thorough timeline of all investigations and the clinical course of the patient is available in Supplementary Figure S2.

## 3 Discussion

In this report, we describe a pediatric case of severe neurological deterioration following COVID-19 where a novel hemizygous loss of function variant in TLR7 was identified. The patient presented with dystonia and finger spasticity that rapidly progressed to dysarthria and hyperreflexia despite his infection being mild and resolved, as reported by his parents. To the best of our knowledge, the variant reported in this case has not yet been described on the gnomAD (6) or ClinVar databases (7).

TLRs comprise a family of dimeric protein receptors primarily located on the surface or inside of immune cells (8). TLRs play a crucial role in the recognition of specific molecular patterns on pathogens and subsequent activation of the innate immune system. Thus, a deficiency or defect in TLR function would suggest an increased risk of infection (9). TLR7, an endosomal receptor, has been demonstrated to recognize single-stranded RNA viruses such as SARS-CoV-2 (10). Upon dimerization, TLR7 initiates a MyD88-dependent cascade magnifying the secretion of pro-inflammatory cytokines such as interleukin-1 (IL-1), IL-23, interferon-alpha (IFN-α, a type I IFN), and IFN-γ (a type II IFN) (11). Although different strands of SARS-COV-2 have been identified, the sequence in which they activate TLR-7 is strikingly similar. This may be attributed to the GU-rich sequences found on the viral antigen (12). Recent evidence highlights an emerging role for TLR7 signaling in the pathogenesis and antiviral response against COVID-19 (10). In parallel, TLR7 deficiency or loss of function is being investigated in cases of severe infection or complications of COVID-19.

In addition to reports of fatigue or generalized weakness following COVID-19, some patients may develop pulmonary (e.g., respiratory failure), cardiovascular (e.g., myocardial infarction), or neurological (e.g., encephalitis or stroke) complications (13–15). Although the exact pathophysiology underlying the severity and long-term effects of COVID-19 is not yet known, specific patient attributes, including genetic factors, may help explain why these complications arise. For instance, in a case–control study of a total 285 participants, it was found that COVID-19 patients with a single nucleotide polymorphism (rs3853839) in TRL7 were at higher risk of poor clinical outcome (5). The G/G genotype, in particular, was associated with the greatest decrease in patient WBC count and increase in serum ferritin and D-dimer levels (*p* < 0.001) suggesting underlying immunopathology. Although no significant difference in mortality between genotypes was found, around 74.0% of patients with the G/G genotype were hospitalized and 22.4% developed cardiac complications (5). Therefore, it was concluded that TLR7 polymorphisms represent a significant genetic risk factor and a possible biomarker for predicting COVID-19 outcomes. Screening for rare TLR7 variants and their use as genomic biomarkers was also recently proposed in a prospective study by Solanich et al. (4). Two out of the fourteen COVID-19 patients included in this study had deleterious TLR7 missense variants (c.644A > G; p.[Asn215Ser]) and (c.2797 T > C; *p*.[Trp933Arg]). Functional testing on peripheral blood mononuclear cells (PBMC) derived from these patients confirmed impaired type I and II IFN responses further suggesting the impact of TLR7 loss of function on COVID-19 outcome (4).

The significant findings and outcomes of reported literature on TLR7 variants in COVID-19 patients are summarized in Table 2. Among those that discussed pediatric patients, only one was found to closely relate to our study: a 7-year-old boy with confirmed COVID-19 infection who developed neurological complications (ataxia-telangiectasia) (20). Both patients were born to consanguineous parents, had no significant family history, and were found to have hemizygous mutations in the TLR7 gene. Brain MRI scans also revealed that both patients had varying degrees of ventricular enlargement and atrophy of brain parenchyma (20). However, several key differences must be highlighted. Compared to our patient, the 7-year-old boy was a known case of hyper IgM syndrome and had an ataxia-telangiectasia mutated (ATM) gene mutation that more likely explains his symptoms. Moreover, despite having an impaired type I IFN response consistent with TLR7 deficiency, his variant (c.1114C > A, p.Leu 372Met) was discovered to be hypomorphic (20). The variant detected in our patient (c.1386_1389dup, p.[His464Ilefs*7]), on the other hand, resulted in truncation of the TLR7 gene and complete loss of function which may allude to the difference in presentation severity.

**Table 2:** Summary of reported literature on COVID-19 patients with detected TLR7 variants or deficiency (ordered by publication year).

Authors	Year	Study design	No. of subjects	Mean age (years)	TLR7 Variant or deficiency	Study outcomes
Garcia et al. (16)	2023	Case series	22 patients: 18 male and 4 female as 17 kindreds from 8 countries	10.9	MyD88 and IRAK4 deficiency which are necessary for TLR7 function	MyD88 and IRAK-4 deficiency impair TLR7-dependent type I IFN production and predispose patients to a higher risk of hypoxemic COVID-19 pneumonia
Gomez et al. (17)	2023	Cross-sectional study	618 patients: 392 male and 226 female	52	TLR7 single nucleotide pleomorphisms (rs179008, rs179009, rs3853839, rs2302267)	TLR7 G/G genotype of the rs3853839 variant was significantly associated with the severity and outcome of critical COVID-19 by an odd ratio of 1.98
Butler-laporte et al. (18)	2022	Exome-wide association study	28,159 patients: 44.1% male and 55.9% female as 21 cohorts from 12 countries	55.6	TLR7 missense variants (unspecified)	TLR7 loss of function increases the risk of severe COVID-19 by up to 13.1-fold in both sexes (despite being located on the X chromosome)
Al-Tamimi et al. (19)	2022	Case series	90 patients: 27 male and 63 female	52.01	TLR7 single nucleotide pleomorphism (rs179008) with A/A, A/T, and T/T genotypes	TLR7 A/A genotype is a risk factor for severe COVID-19
Abolhassani et al. (20)	2022	Case report	1 patient: single male	7	TLR7 hemizygous deleterious variant (c.1114C > A, p.Leu372Me)	Reports severe COVID-19 pneumonia with ataxia and telangiectasia in a pediatric patient Brain MRI showed fusiform gyrus and cerebellar atrophy, 4^th^ ventricle enlargement, subarachnoid fluid
Alseoudy et al. (21)	2022	Case–control study	136 case patients: 84 male and 52 female 100 control patients: 40 male and 60 female	61.33	TLR7 single nucleotide pleomorphism (rs179008) with A/A, A/T, and T/T genotypes	TLR7 A/A genotype increases the risk of COVID-19 pneumonia in males by 49.2-fold compared to females No significant association was found between SNP genotype and COVID-19 pneumonia outcome
El-Hefnawy et al. (5)	2022	Case–control study	150 case patients: 81 male and 69 female 125 control patients: 80 male and 55 female	38.07	TLR7 single nucleotide pleomorphism (rs3853839) with G/G, G/C, and C/C genotypes	TLR7 G/G phenotype was associated with higher levels of serum TLR7 mRNA expression, severe pneumonia, and poor prognosis 61.8% of patients with G/G phenotype suffered respiratory failure with 4 cases of mortality TLR7 G/G phenotype was proposed as a predictive biomarker for COVID-19 severity and outcome
Pessoa et al. (22)	2021	Case report	3 patients: 1 male and 2 female	7.5	TLR7 single nucleotide pleomorphism (rs179008) with A/T genotype	All three children have sickle cell disease and developed complications during hospitalization following severe COVID-19
Mantovani et al. (23)	2021	Brief communication	Unspecified, selected from a cohort used in Fallerini et al.	< 60	TLR7 new deleterious variant predicted by *in silico* analysis (Asp41Glu)	TLR7 loss of function variants (but not hypomorphic or hypofunctional variants) decrease mRNA expression of RSAD2, ACOD1, and IFIT2 that mediate TLR7 signaling causing significant impairment of the antiviral response
Fallerini et al. (24)	2021	Nested case–control study	239 case patients: all male 77 control patients: all male	< 60	TLR7 single nucleotide pleomorphisms (rs189681811, rs147244662, rs149314023, rs200146658, rs5743781)	Patients had impaired type I and II IFN responses TLR7 loss of function variants increase susceptibility to severe COVID-19 as they were detected in 2.1% of critical cases but never in asymptomatic patients
Solanich et al. (4)	2021	Prospective study	14 patients: all male	38	TLR7 deleterious missense variants (c.644A > G; p.[Asn215Ser]) and (c.2797 T > C; p.[Trp933Arg])	TLR7 variants and impaired type I and II IFN responses were detected in 2 out of 14 severely affected patients Circulating 25-hydroxy vitamin D levels decline with age and may contribute to deterioration in TLR7 function and increase susceptibility to severe COVID-19 Young males at-risk of developing severe COVID-19 may benefit from pre-symptomatic genetic screening for TLR7
Asano et al. (25)	2021	Case series	17 patients: all male as 16 kindreds selected from a greater cohort of 1,202 patients with unexplained critical COVID-19 pneumonia	36.7	TLR7 deleterious variants (L134P/Y, N158Tfs11*/Y, L227fs*/Y, D244Y/Y, F310L/Y, L372M, I505T/Y, H630Y/Y, I657T/Y, F670Lfs*8, K684*/Y, P715S/Y, H781L/Y, L988S/Y, M854I;L988S/Y)	TLR7 deficiency due to missense or loss of function variants was found to be a highly penetrant genetic factor of life-threatening COVID-19 pneumonia From the general population, it is estimated that 1.8% of males below 60 years of age have X-linked recessive TLR7 deficiency with an allele frequency < 6.5×10^−4^
Pessoa et al. (26)	2021	Case report	1 patient: single male	5	TLR7 single nucleotide pleomorphisms (rs179008) with A/T genotype	Reports fever, hepatitis, alcoholic stool, and jaundice following COVID-19 infection in a pediatric patient
Van der Made et al. (27)	2020	Case series	4 patients: all male as two unrelated pairs of brothers	26	TLR7 four-nucleotide deletion (c.2129_2132del; p.[Gln710Argfs*18]) and missense variant (c.2383G > T; p.[Val795Phe])	Patients had impaired type I and II IFN responses due to defective downstream TLR7 signaling as evidenced by decreased expression of IRF7, IFNB1, and ISG15 mRNA

This study has several limitations. First, no PBMC sample was available to test and confirm the functional impact of the novel TLR7 mutation detected in our patient. However, the frameshift variant we observed resulted in a downstream premature stop codon of 7 amino acids removing more than 10% of the transcript (last 10% transcript position 12,888,342). The truncated region is critical to proper TLR7 function, and its loss is a known mechanism of X-linked COVID-19-related immunodeficiency-74 (28). Moreover, this frameshift mutation was classified as a likely pathogenic (class 2) null variant based on American College of Medical Genetics and Genomics (ACMG) recommendations (29), further suggesting that the genetic findings in our patient are unlikely due to chance. Second, the pathogenicity or clinical significance of MEFV, SCN5A, and STAT3 were not entirely determined. Although they do not appear responsible for the patient’s initial presentation or neurological deterioration, firm conclusions on the degree of their involvement cannot be drawn. Lastly, more studies are needed to validate the results in this case. Our current understanding of the association and critical role of TLR7 in COVID-19 is still expanding and future research may help shed light on possible applications in preventive screening or targeted therapy.

## 4 Conclusion

In summary, we have identified a novel frameshift null variant of the X chromosomal TLR7 gene (c.1386_1389dup, p.[His464Ilefs*7]) in a pediatric patient with neurological deterioration following COVID-19 infection. Previous reports on COVID-19 patients with TLR7 variants conclude that TLR7 deficiency is an important risk factor for disease severity. However, our preliminary findings also suggest TLR7 loss of function may contribute to neurological complications post-COVID-19, especially in young previously healthy patients.

## Data availability statement

The original contributions presented in the study are included in the article/supplementary material, further inquiries can be directed to the corresponding author.

## Ethics statement

The studies involving humans were approved by Prince Sultan Military Medical City (PSMMC) IRB Committee (IRB-Psmmc-934). The studies were conducted in accordance with the local legislation and institutional requirements. The human samples used in this study were acquired from a by– product of routine care or industry. Written informed consent for participation was not required from the participants or the participants’ legal guardians/next of kin in accordance with the national legislation and institutional requirements. Written informed consent was obtained from the minor(s)’ legal guardian/next of kin for the publication of any potentially identifiable images or data included in this article.

## Author contributions

ANE: Investigation, Methodology, Visualization, Writing – original draft, Writing – review & editing. MA-R: Investigation, Methodology, Writing – original draft. FP-Z: Investigation, Methodology, Writing – original draft. KH: Project administration, Supervision, Writing – review & editing. AA: Project administration, Supervision, Writing – review & editing.
