# Supplementary material for: Novel TLR7 hemizygous variant in post-COVID-19 neurological deterioration: a case report with literature review
Source: Front Neurol. 2023 Nov 29;14:1268035. doi: 10.3389/fneur.2023.1268035 (PMC10716429; doi:10.3389/fneur.2023.1268035)
Supplement: Supplementary file 1 [file Image_1.pdf]

Supplementary Figure 1

Novel TLR7 Hemizygous Variant in Post-COVID-19 Neurological Deterioration: a case report with literature review

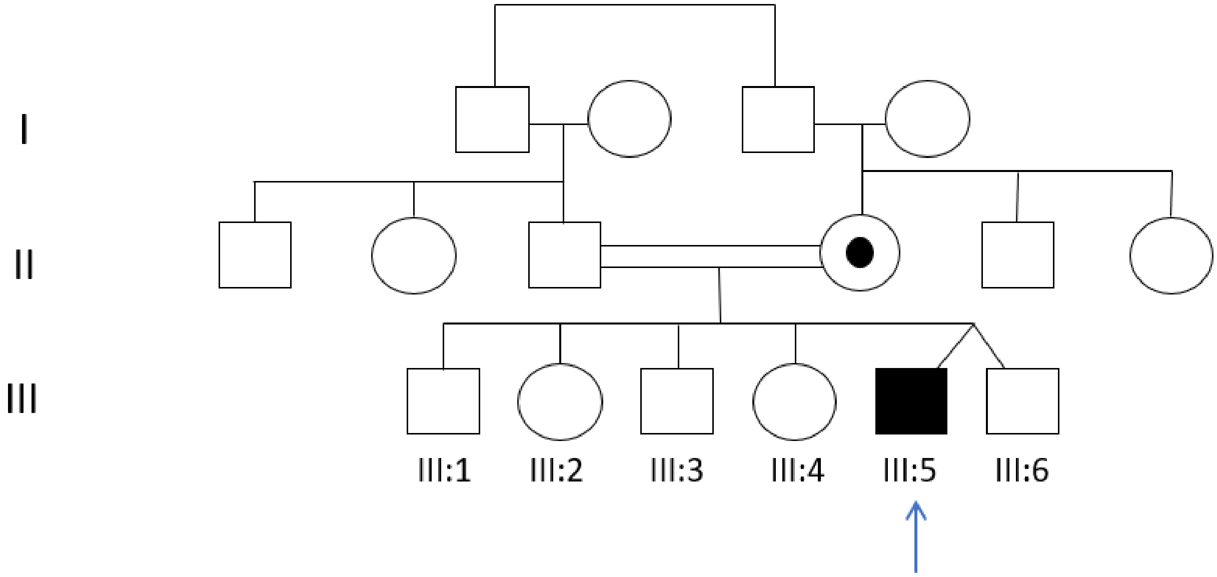

Supplementary Figure 1. Patient family pedigree with affected index
