# Supplementary material for: Novel TLR7 hemizygous variant in post-COVID-19 neurological deterioration: a case report with literature review
Source: Front Neurol. 2023 Nov 29;14:1268035. doi: 10.3389/fneur.2023.1268035 (PMC10716429; doi:10.3389/fneur.2023.1268035)
Supplement: Supplementary file 3 [file Table_1.docx]

Supplementary Table 1

Novel TLR7 Hemizygous Variant in Post-COVID-19 Neurological Deterioration: a case report with literature review

**Authors:** Ahmed Noor Eddin*, Mohammed Al-Rimawi, Feham Peer-Zada, Khalid Hundallah, Amal Alhashem

*** Correspondence:** Ahmed Noor Eddin: [neddin.ahmed@gmail.com](mailto:neddin.ahmed@gmail.com)

| **Variable** | **Test result** |
| --- | --- |
| dsDNA Antibody | Negative |
| Antinuclear Antibody (ANA) | Negative |
| Antineutrophil Cytoplasmic Antibody (cANCA) | Negative |
| Antineutrophil Cytoplasmic Antibody (pANCA) | Negative |
| Anti-Myeloperoxidase Antibody (anti-MPO) | 1.39 (normal) |
| Anti-Proteinase 3 Antibody (anti-PR3) | 1.37 (normal) |
| Glutamine Receptor Antibody (NMDA type) | Negative |
| Aquaporin 4 Antibody (NMO) | Negative |
| Anti-Glutamic Acid Decarboxylase (anti-GAD) | 35.0 (normal) |
| Anti-Islet Cell Antibody | Negative |
| Anti-Tyrosine Phosphatase-related Islet Antigen 2 (anti-IA2) | Negative |

**Supplementary Table 1.** Autoimmune Serology
