# Supplementary material for: Novel TLR7 hemizygous variant in post-COVID-19 neurological deterioration: a case report with literature review
Source: Front Neurol. 2023 Nov 29;14:1268035. doi: 10.3389/fneur.2023.1268035 (PMC10716429; doi:10.3389/fneur.2023.1268035)
Supplement: Supplementary file 4 [file Table_2.docx]

Supplementary Table 2

Novel TLR7 Hemizygous Variant in Post-COVID-19 Neurological Deterioration: a case report with literature review

**Authors:** Ahmed Noor Eddin*, Mohammed Al-Rimawi, Feham Peer-Zada, Khalid Hundallah, Amal Alhashem

*** Correspondence:** Ahmed Noor Eddin: [neddin.ahmed@gmail.com](mailto:neddin.ahmed@gmail.com)

| **Variable** | **Reference Range** | **Test result** |
| --- | --- | --- |
| Blood IgA (g/L) | 0.8-3.0 | 1.28 |
| Blood IgG (g/l) | 6.0-16.0 | 8.52 |
| Blood IgM (g/L) | 0.4-2.5 | 1.14 |
| Serum IgG (g/L) | 6.0-16.0 | 8.43 |
| Serum Albumin (g/L) | 34-54 | 26.30 (Low) |
| CSF Turbidity & Color | - | Clear and Colorless |
| CSF RBC count | 0.0 | 0.0 |
| CSF WBC count | <3 | 0.0 |
| CSF Glucose (mmol/L) | 2.5-3.5 | 3.5 |
| CSF Total Protein (mmol/L) | 0.23-035 | 0.37 (High) |
| CSF IgG (mg/L) | 0.0-0.045 | 0.135 (High) |
| CSF Albumin (mg/L) | 0.05-0.34 | 0.158 |
| CSF IgG/Albumin Ratio | <0.28 | 2.67 (High) |
| CSF Albumin Oligoclonal Band | - | Positive |

**Supplementary Table 2.** Patient CSF and Serum Profile (significant findings are highlighted in red)
